# Evolution and transmission of antibiotic resistance is driven by Beijing lineage *Mycobacterium tuberculosis* in Vietnam

**DOI:** 10.1128/spectrum.02562-23

**Published:** 2023-11-16

**Authors:** Matthew Silcocks, Xuling Chang, Nguyen Thuy Thuong Thuong, Youwen Qin, Dang Thi Minh Ha, Phan Vuong Khac Thai, Srinivasan Vijay, Do Dang Anh Thu, Vu Thi Ngoc Ha, Hoang Ngoc Nhung, Nguyen Huu Lan, Nguyen Thi Quynh Nhu, David Edwards, Artika Nath, Kym Pham, Nguyen Duc Bang, Tran Thi Hong Chau, Guy Thwaites, A. Dorothee Heemskerk, Chiea Chuen Khor, Yik Ying Teo, Michael Inouye, Rick Twee-Hee Ong, Maxine Caws, Kathryn E. Holt, Sarah J. Dunstan

**Affiliations:** 1 Department of Infectious Diseases, University of Melbourne at the Peter Doherty Institute for Infection and Immunity, Parkville, Victoria, Australia; 2 Department of Paediatrics, Yong Loo Lin School of Medicine, National University of Singapore, , Singapore; 3 Khoo Teck Puat–National University Children’s Medical Institute, National University Health System, Singapore; 4 Oxford University Clinical Research Unit, Hospital for Tropical Diseases, District 5, Ho Chi Minh City, Vietnam; 5 Nuffield Department of Medicine, Centre for Tropical Medicine and Global Health, University of Oxford, Oxford, United Kingdom; 6 Cambridge Baker Systems Genomics Initiative, Baker Heart and Diabetes Institute, Melbourne, Victoria, Australia; 7 School of BioSciences, The University of Melbourne, Melbourne, Victoria, Australia; 8 Pham Ngoc Thach Hospital for TB and Lung Disease, District 5, Ho Chi Minh City, Vietnam; 9 Theoretical Microbial Ecology, Friedrich Schiller University Jena, Jena, Germany; 10 Department of Infectious Diseases, Central Clinical School, Monash University, Melbourne, Victoria, Australia; 11 Department of Clinical Pathology, The University of Melbourne, Melbourne, Victoria, Australia; 12 Hospital for Tropical Diseases, District 5, Ho Chi Minh City, Vietnam; 13 Department of Medical Microbiology and Infection Prevention, Amsterdam University Medical Centre, Amsterdam, Netherlands; 14 Genome Institute of Singapore, Singapore; 15 Saw Swee Hock School of Public Health, National University of Singapore, Singapore; 16 Department of Public Health and Primary Care, Cambridge Baker Systems Genomics Initiative, University of Cambridge, Cambridge, United Kingdom; 17 Liverpool School of Tropical Medicine, Liverpool, United Kingdom; 18 Birat Nepal Medical Trust, Kathmandu, Nepal; 19 Department of Infection Biology, London School of Hygiene & Tropical Medicine, London, United Kingdom; CNRS - University of Toulouse, Toulouse, France

**Keywords:** antimicrobial resistance, pathogen genomics, *Mycobacterium tuberculosis*

## Abstract

A previous investigation has elucidated the landscape of *Mtb* genomic diversity and transmission dynamics in Ho Chi Minh City, Vietnam. Here, we expand the scope of this survey by adding a substantial number of additional genomes (total sample size: 2,542) and phenotypic drug susceptibility data for the majority of isolates. We aim to explore the prevalence and evolutionary dynamics of drug resistance and our ability to predict drug resistance from sequencing data. Among isolates tested phenotypically against first-line drugs, we observed high rates of streptomycin [STR, 37.7% (*N* = 573/1,520)] and isoniazid resistance [INH, 25.7% (*N* = 459/1,786)] and lower rates of resistance to rifampicin [RIF, 4.9% (*N* = 87/1,786)] and ethambutol [EMB, 4.2% (*N* = 75/1,785)]. Relative to global benchmarks, resistance to STR and INH was predicted accurately when applying the TB-Profiler algorithm to whole genome sequencing data (sensitivities of 0.81 and 0.87, respectively), while resistance to RIF and EMB was predicted relatively poorly (sensitivities of 0.70 and 0.44, respectively). Exploring the evolution of drug resistance revealed the main phylogenetic lineages to display differing dynamics and tendencies to evolve resistance via mutations in certain genes. The Beijing sublineage L2.2.1 was found to acquire *de novo* resistance mutations more frequently than isolates from other lineages and to suffer no apparent fitness cost acting to impede the transmission of resistance. Mutations conferring resistance to INH and STR arose earlier, on average, than those conferring resistance to RIF and are now more widespread across the phylogeny. The high prevalence of “background” INH resistance, combined with high rates of RIF mono-resistance (20.7%, *N* = 18/87), suggests that rapid assays for INH resistance will be valuable in this setting. These tests will allow the detection of INH mono-resistance and will allow multi-drug-resistant isolates to be distinguished from isolates with RIF mono-resistance.

**IMPORTANCE:**

Drug-resistant tuberculosis (TB) infection is a growing and potent concern, and combating it will be necessary to achieve the WHO’s goal of a 95% reduction in TB deaths by 2035. While prior studies have explored the evolution and spread of drug resistance, we still lack a clear understanding of the fitness costs (if any) imposed by resistance-conferring mutations and the role that *Mtb* genetic lineage plays in determining the likelihood of resistance evolution. This study offers insight into these questions by assessing the dynamics of resistance evolution in a high-burden Southeast Asian setting with a diverse lineage composition. It demonstrates that there are clear lineage-specific differences in the dynamics of resistance acquisition and transmission and shows that different lineages evolve resistance via characteristic mutational pathways.

## INTRODUCTION

Tuberculosis (TB) remains a global epidemic with one quarter of the world’s population estimated to be infected. The impact of the COVID-19 pandemic on essential TB services has reversed years of progress, with the number of newly diagnosed TB patients falling to 5.8 million in 2020, much less than the estimated 10 million who developed TB (1). An increase in TB deaths in 2020 was also estimated (1.3 million in HIV negative, and 214,000 in HIV positive) due largely to a reduction in the number of people treated for drug-resistant TB (1). Geographically, the burden of disease lies mainly in Southeast Asia (44% of TB cases in 2018) (2), with 86% of new TB cases worldwide being reported from 30 high TB burden countries in 2020 (1).

Vietnam is a high TB burden country, designated by its high number of incident TB and multi-drug-resistant (MDR) TB cases (3). In 2018, 174,000 and 8,600 people in Vietnam fell ill with TB and drug-resistant TB, respectively. Of the patients with drug-resistant TB, only 36.3% were laboratory confirmed, and 36.2% started on second-line treatment.

Although the COVID-19 pandemic has set back recent progress, steps toward the WHO END-TB targets have been made. To be able to achieve a 95% reduction of TB deaths by 2035 (4), modern technologies must be embraced to find innovative ways to accelerate TB control and elimination. One such technology is genomic sequencing, which offers a myriad of opportunities for innovation in diagnostics, treatment, prevention, and control of TB.

Genotype data, for example, can be used to predict drug resistance in *Mtb* isolates by querying databases of confirmed or suspected resistance-conferring variants (5 – 7). This approach provides an efficient alternative to traditional phenotypic methods which are prone to human error and contamination and require time-consuming bacterial culturing (7 – 9). This technology has the potential to reduce the probability of misassignment of drugs to patients infected with resistant *Mtb* (compared to standardized drug regimens) and to reduce the time before patients receive effective treatment, potentially leading to more favorable treatment outcomes (10).

Despite the promise of genotype-based drug resistance prediction, its accuracy has been shown to vary according to human population, *Mtb* lineage, type of drug, and the prediction protocol used (11). A more comprehensive understanding of the effect that these factors have on prediction accuracy and the levels of accuracy which are attainable across diverse cohorts is necessary prior to implementation of these tests in all clinical settings.

Genomics also provides insight into the emergence, transmission, and overall dynamics of drug resistance in *Mtb* via the use of a phylogenetic toolkit (12 – 17). Prior studies have produced time-calibrated phylogenies to date the acquisition of drug resistance in *Mtb* lineages (12, 18) and explored their expansion through time (14). Others have used models of ancestral sequence reconstruction (13, 17, 18) and single nucleotide polymorphism (SNP) clustering methods (16, 19) to compare the rates of acquired (*de novo*) versus primary (i.e., transmitted) drug resistance within a population.

While these studies naturally vary in their scope, cohort size, geographic scale, and setting, their results have highlighted similar trends in drug resistance evolution. They show that the global drug resistance burden has arisen through both the *de novo* acquisition of drug resistance and through the transmission of drug-resistant *Mtb* to new hosts (13, 16, 19 – 21). They also show consistent trends in the order of drug resistance acquisition, with isoniazid (INH) resistance resistance generally arising prior to rifampicin (RIF) resistance and being more deeply rooted in the phylogeny (17, 18, 20). Finally, when characterizing *Mtb* lineage diversity, these studies have implicated Beijing lineage isolates in many outbreaks (13, 16, 19).

Here, we apply these tools to investigate resistance evolution in a Southeast Asian context, using a collection of genomes from Ho Chi Minh City (HCMC), Vietnam, which has a high frequency of TB, drug resistance, and the Beijing lineage. In a prior study, we collected whole genome sequencing (WGS) data from 1,635 *Mtb* isolates from HCMC and explored the lineage composition, signals of homoplasy, and trends in transmission dynamics. This analysis highlighted the threat of the L2.2.1 sublineage, which was associated with younger patient age, cross-border spread, and high rates of transmission within the population. In addition to substantially increasing the number of available genomes, this investigation contributes phenotypic drug resistance data for the majority of isolates. Our objectives here are to gauge the frequency of drug resistance, assess trends in its evolution and transmission dynamics, and measure our ability to predict it using WGS data.

## RESULTS

### Patient clinical characteristics and genetic diversity of *Mtb* isolates

To characterize the diversity and drug resistance of *Mtb* in HCMC, Vietnam, we analyzed the genomic sequences of *N* = 2,542 isolates (post quality filtering) cultured from TB patients between 2001 and 2013. A subset of these genomes (*N* = 1,627), derived from pulmonary TB (PTB) patients, were published previously (22). Here, these are supplemented with an additional *N* = 914 novel genomes, comprising *N* = 747 from PTB cases and 167 from tuberculous meningitis (TBM) cases (23, 24). A single lineage 5 genome was included as an outgroup for phylogenetic analyses. PTB patients were sputum culture positive and ≥18 years old (25), whereas TBM patients were cerebral spinal fluid culture positive and ≥15 years old (23, 24). All TB patients were human immunodeficiency virus (HIV) negative.

**Fig 1:**
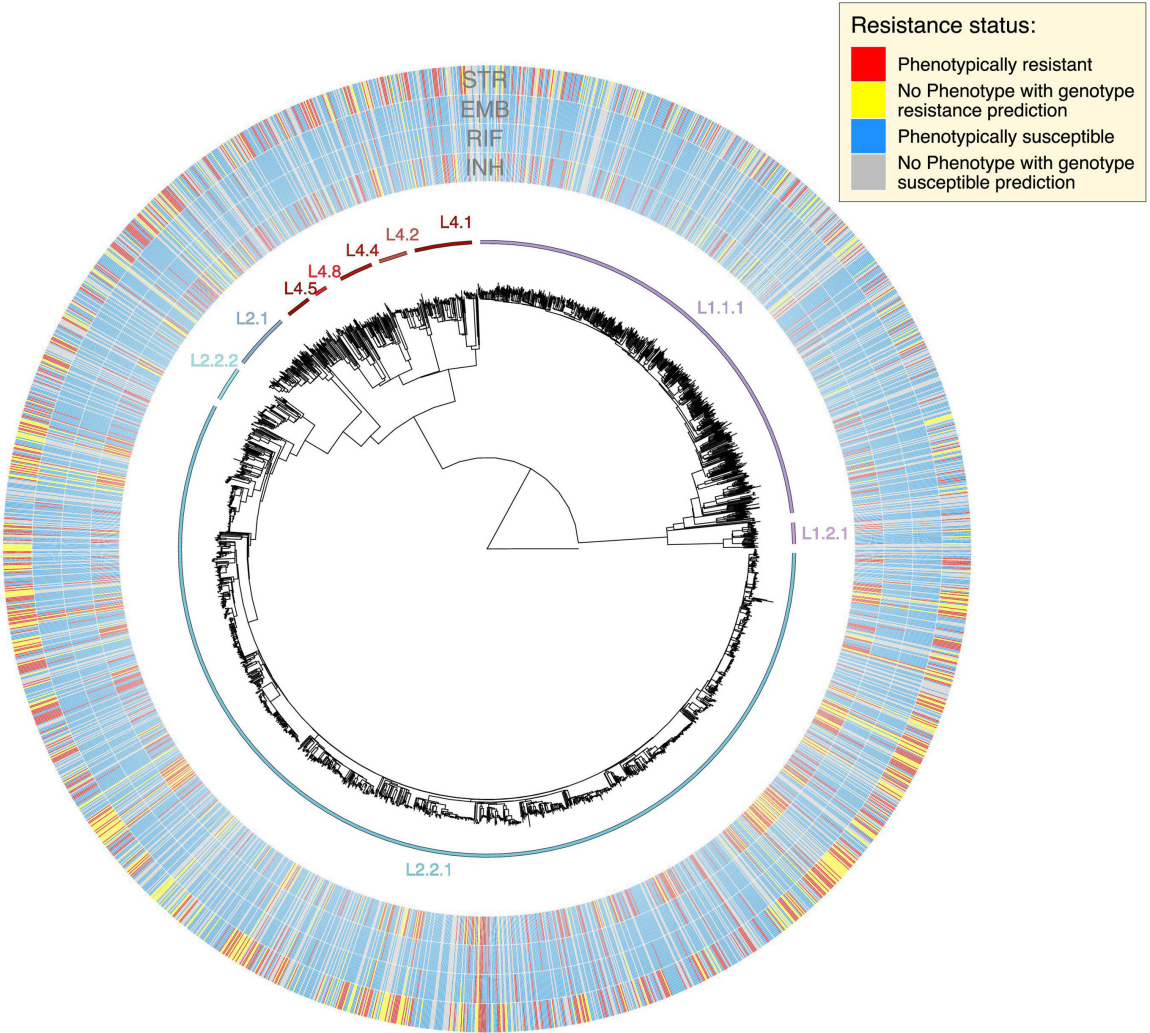
Phylogeny of 2,542 *Mtb* genomes, with associated phenotypic and genotypic drug resistance data for four first-line drugs [moving outwards: INH, RIF, ethambutol (EMB), and streptomycin (STR), respectively]. For all samples with gold standard phenotypic drug susceptibility testing (DST) data (*N* = 1,781), red denotes resistant isolates, and blue denotes susceptible isolates. For all isolates without phenotypic DST data, yellow denotes genotypically predicted resistant isolates, and gray denotes genotypically predicted susceptible isolates. As noted elsewhere, phenotypic resistance and genotypic-based resistance prediction statuses were not perfectly correlated. Of all isolates predicted to be resistant using genotype data, 92.6%, 79.2%, 40.2%, and 86.5% were phenotypically resistant for INH, RIF, EMB, and STR, respectively (positive predictive value; Fig. 2). Of all isolates predicted to be susceptible using genotype data, 95.6%, 98.5%, 97.6%, and 89.2% were phenotypically susceptible for these same four drugs (negative predictive value; Fig. 2).

Our analysis of the genetic diversity of *Mtb* lineages and clinical characteristics of patients reiterated prior findings based on a subset of this data set (22). The majority of all TB cases were males (71.5% male, 28.5% female), with a median (interquartile) age of 39 (26–49) years. The East Asian lineage 2 was the most prevalent *Mtb* lineage (*N* = 1,615, 63.6%), followed by the East African-Indian Ocean lineage 1 (*N* = 649, 25.5%), the European-American lineage 4 (*N* = 275, 10.8%), and the Delhi Central Asian lineage 3 (*N* = 2, 0.1%; Fig. 1). Lineage 2 and sublineage 2.2.1 were more prevalent in female patients (lineage 2; 67.4% of females vs 62.0% of males; *P* = 0.010; Table 1), [sublineage 2.2.1; 62.3% of females vs 55.8% of males; *P* = 0.005 (when excluding other L2 isolates from test)], while lineage 1 showed a higher prevalence amongst males (27.3% of males vs 21.1% of females; *P* = 0.001; Table 1). Lineage 2 was significantly associated with younger people (median, 37 years), and lineage 1, with older people (median, 43 years; *P* = 8.36 × 10^−8^).

**TABLE 1:** Lineage distribution by sex and clinical phenotype. A dash indicates a count of zero for that cell.

	Lineage						
	1	2.1	2.2.1	2.2 (other than 2.2.1)	3	4	Total
Sex							
Male	496 (27.3%)	68 (3.7%)	1,014 (55.8%)	44 (2.4%)	1 (0.1%)	193 (10.6%)	1,816 (71.5%)
Female	153 (21.1%)	18 (2.5%)	452 (62.3%)	19 (2.6%)	1 (0.2%)	82 (11.3%)	725 (28.5%)
Clinical phenotype							
PTB	581 (24.5%)	82 (3.50%)	1,389 (58.5%)	57 (2.4%)	2 (0.1%)	263 (11.1%)	2,374 (93.4%)
TBM	68 (40.7%)	4 (2.4%)	77 (46.1%)	6 (3.6%)	–	12 (7.2%)	167 (6.6%)
All TB	649 (25.5%)	86 (3.4%)	1,466 (57.7%)	63 (2.5%)	2 (0.1%)	275 (10.8%)	2,541

*Mtb* sublineage distributions also resembled those of Holt et al. (22), although via recent studies, we now benefit from a greater understanding of the geographic distribution of each of these (26 – 28). Lineage 1 isolates belonged predominantly to sublineages 1.1.1.1 (*N* = 525) and 1.1.1 (*N* = 83), which are most frequent in Mainland Southeast Asia (26, 27); (Fig. 1), and L1.2.1 (*N* = 33), which is most frequent in Island Southeast Asia (26, 28). The three remaining L1 sublineages were observed either once or never (L1.1.3, *N* = 1; L1.2.2, *N* = 1; L1.1.2, *N* = 0), supporting the deep geographic structure of the lineage.

Isolates from lineage 2 could be partitioned into both the “Proto-Beijing” (L2.1; *N* = 86) and Beijing (L2.2; *N* = 1,529) sublineages (29) (Fig. 1). Beijing lineage isolates belonged to both L2.2.1 (*N* = 1,446), which has a wide global distribution (22), as well as the less prevalent L2.2.2 (*N* = 55) (30). We documented a wide array of L4 sublineages, consistent with a history of repeated introductions into Southeast Asia, as inferred by Holt et al. (22). The most frequent L4 sublineages were 4.1, 4.2, 4.4, and 4.5.

### Phenotypic drug resistance of PTB isolates

Phenotypic drug susceptibility testing was performed for a subset of the sequenced isolates (*N* = 1,786). We assessed phenotypic resistance to three first-line drugs [INH, RIF, and ethambutol (EMB)] using mycobacterial growth indicator tubes (MGIT) (*N* = 1,786) and also tested a subset of these isolates using UKMYC5 (*N* = 267). Isolates were deemed resistant to an antimicrobial if they were classified as resistant by one or both methods. In total, 459 of 1,786 (25.7%) isolates were resistant to INH; 87 of 1,786 (4.9%), to RIF; and 75 of 1,785 (4.2%), to EMB. In total, 3.9% (*N* = 69/1,786) were MDR (INH and RIF resistant; Table 2), and 20.7% (*N* = 18/87) of RIF-resistant isolates were INH sensitive. Streptomycin (STR) resistance, which was tested by MGIT only, was identified in 37.7% (573/1,520) of the tested isolates. Although sample sizes were relatively low, rates of resistance to a number of second-line drugs were found to be high within a global context [para-aminosalicylic acid (PAS) (29.6%, *N* = 79/267), amikacin (AMI) (15.4%, *N* = 41/267), and moxifloxacin (MXF) (21.3%, *N* = 57/267); Table 2].

**TABLE 2:** Antimicrobial resistance by lineage^*a*^

Drug resistance in PTB	1	2.1	2.2.1	2.2 (other than 2.2.1)	4	Total resistant	Total tested
MDR							
Isoniazid/rifampicin	7 (10.1%)	4 (5.8%)	53 (76.8%)	2 (2.9%)	3 (4.3%)	69 (3.9%)	1,786
Antimicrobial (DST by either)							
Isoniazid	74 (16.1%)	18 (3.9%)	311 (67.8%)	6 (1.3%)	50 (10.9%)	459 (25.7%)	1,786
Rifampicin	11 (12.6%)	5 (5.7%)	64 (73.6%)	2 (2.3%)	5 (5.7%)	87 (4.9%)	1,786
Ethambutol	7 (9.3%)	2 (2.7%)	58 (77.3%)	1 (1.3%)	7 (9.3%)	75 (4.2%)	1,785
Antimicrobial (DST by MGIT)							
Streptomycin	78 (13.6%)	29 (5.1%)	391 (68.2%)	11 (1.92%)	64 (11.2%)	573 (37.7%)	1,520
Antimicrobial (DST by UKMYC5)							
Bedaquiline (BDQ ≥1)	–	–	–	1 (100%)	–	1 (0.4%)	267
Kanamicin (KAN ≥2.5)	1 (6.7%)	2 (13.3%)	5 (33.3%)	1 (6.7%)	6 (40.0%)	15 (5.6%)	267
Ethionamide (ETH ≥5)	12 (54.5%)	–	10 (45.5%)	–	–	22 (8.2%)	267
Amikacin (AMI ≥1)	10 (24.4%)	1 (2.4%)	22 (53.7%)	4 (9.8%)	4 (9.8%)	41 (15.4%)	267
Levofloxacin (LEV ≥1)	8 (25.8%)	1 (3.2%)	18 (58.1%)	2 (6.5%)	2 (6.5%)	31 (11.6%)	267
Moxifloxacin (MXF ≥0.5)	10 (17.5%)	1 (1.8%)	33 (57.9%)	5 (8.8%)	8 (14.0%)	57 (21.3%)	267
Delamanid (DLM ≥0.12)	–	–	4 (80.0%)	1 (20.0%)	–	5 (1.9%)	267
Linezolid (LZD ≥1)	14 (43.8%)	–	13 (40.6%)	–	5 (15.6%)	32 (12.0%)	267
Ceftazadime (CFZ ≥1)	2lkl, bbn (28.6%)	–	3 (42.9%)	–	2 (28.6%)	7 (2.6%)	267
Rifabutin (RFB ≥1)	–	1 (20.0%)	4 (80.0%)	–	–	5 (1.9%)	267
Para-aminosalicylic acid (PAS ≥4)	31 (39.2%)	2 (2.5%)	36 (45.6%)	5 (6.3%)	5 (6.3%)	79 (29.6%)	267

^*a*^ A dash indicates a count of zero for that cell.

We noted that rates of resistance to all first-line drugs were higher for isolates belonging to lineage 2 when compared to lineages 1 and 4 [Pearson’s χ^2^ test; INH (*P* = 9.78 × 10^−6^), RIF (*P* = 8.67×10^−4^), EMB (*P* = 0.003), and STR (*P* = 2.83 × 10^−12^); Table 2], a finding consistent with several prior studies (13, 16, 31, 32). This trend persists when restricting the comparison to isolates from the L2.2.1 sublineage [INH (*P* = 2.48×10^−6^), RIF (*P* = 0.001), EMB (*P* = 0.001), and STR (*P* = 3.63×10^−12^); Table 2], a sublineage which is associated with enhanced transmissibility relative to other L2 sublineages (22).

### Prediction of drug resistance using genotype data

**Fig 2:**
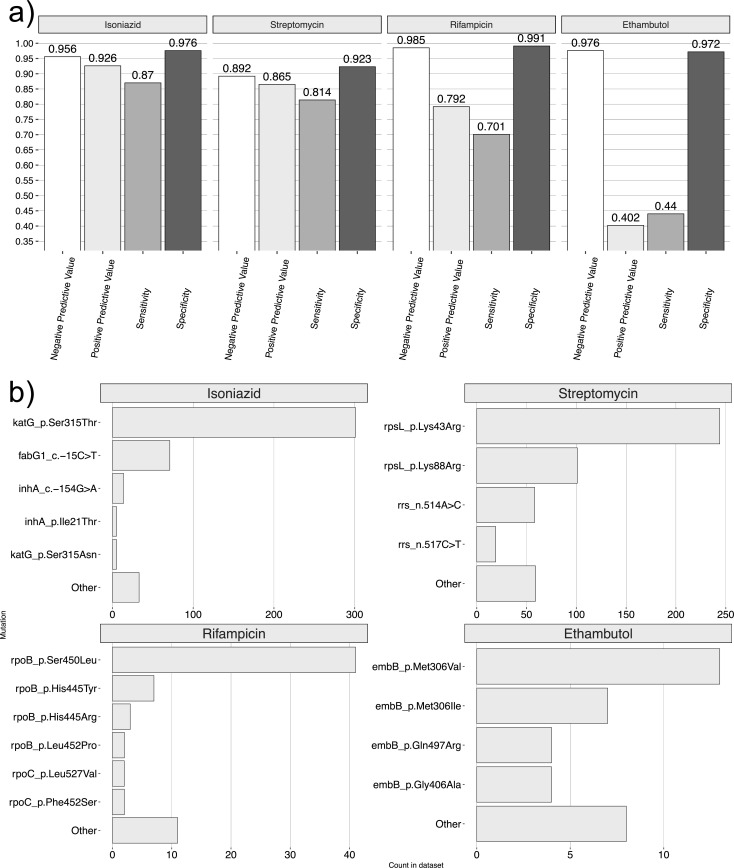
(a) Sensitivity, specificity, and positive and negative predictive values for four first-line drugs inferred using TB-Profiler. (b) The count of resistance-conferring mutations/amino acid substitutions in phenotypically resistant isolates.

We assessed our ability to correctly predict drug resistance using genotype data and found it to vary according to the drug and the phenotype (either “resistant” or “susceptible”) being predicted. We analyzed raw sequencing data with TB-Profiler (33) and calculated sensitivity and specificity metrics using standard approaches (34). Sensitivity, which measures the proportion of phenotypically resistant strains correctly predicted to be resistant, for first-line drugs was 0.87, 0.70, 0.44, and 0.81 for INH, RIF, EMB, and STR, respectively (Fig. 2a). Specificity, which measures the proportion of phenotypically susceptible strains correctly predicted to be susceptible, was 0.98, 0.99, 0.97, and 0.92 for these same four drugs, respectively (Fig. 2a).

Positive predictive values (PPVs), which measure the proportion of isolates with a resistance prediction which were phenotypically resistant, were 0.93, 0.79, 0.40, and 0.87 for INH, RIF, EMB, and STR, respectively (Fig. 2a). Negative predictive values, which measure the proportion of isolates with a susceptible prediction which were phenotypically susceptible, were 0.96, 0.98, 0.98, and 0.89 (Fig. 2a).

We observed differences in the sensitivity of resistance prediction between lineages (Fig. S1), a finding consistent with the tendency of lineages to evolve resistance via certain pathways (discussed subsequently). In particular, the sensitivity of STR resistance prediction among lineage 2 isolates (0.89) exceeded that of L1 (0.64) and L4 (0.52) isolates (Fig. S1). The low number of isolates with resistant phenotypes for RIF and EMB among lineages 1 and 4 (all ≤11 isolates) precluded us from drawing strong conclusions about lineage-specific sensitivities for these drugs.

The frequency distribution of mutations explaining resistance to first-line drugs (Fig. 2b) was skewed toward a number of commonly reported variants (35). In particular, the *katG*-Ser315Thr substitution was the most frequently observed mutation in INH-resistant cases (*N* = 299/459, 65.1% of resistant isolates), followed by *fabG1*-15C > T (*N* = 71/459, 15.5%). RIF resistance was most commonly explained by *rpoB*-Ser450Leu (*N* = 41/87, 47.1%), and STR resistance, by *rpsL*-Lys43Arg (*N* = 243/573, 42.4%) and *rpsL*-Lys88Arg (*N* = 100/573, 17.5%). EMB displayed a wider spectrum of resistance-conferring mutations, with the most frequent being *embB*-Met306Val (*N* = 13/75, 17.3%) and *embB*-Met306Ile (*N* = 7/75, 9.3%). Importantly, in the majority of cases (*N* = 42/75, 56%), resistance to EMB was not explained by any known markers.

Resistance prediction for second-line drugs was overall poor, suggesting that there may be undiscovered resistance variants in this population [sensitivity values: ethionamide (ETH) = 0.41 (*N* = 22 resistant isolates), AMI = 0 (*N* = 41), levofloxacin (LEV) = 0.07 (*N* = 31), MXF = 0.04 (*N* = 57), linezolid (LZD) = 0 (*N* = 32), kanamicin (KAN) = 0 (*N* = 15), and PAS = 0.05 (*N* = 79)]. Sensitivity values for drugs with fewer than 10 resistant isolates are provided in the Supplementary Material (Table S1). Positive predictive values for these drugs were generally quite high; however, suggesting known resistance markers will be specific for predicting drug resistance in this population [PPVs for drugs with at least one resistance mutation: LEV = 1.0 (*N* = 2 resistance mutations), MXF = 1.0 (*N* = 2), delamanid (DLM) = 0.8 (*N* = 5), ETH = 0.69 (*N* = 13), and PAS = 1.0 (*N* = 4)].

To assist screens for second-line drug resistance mutations, we scanned for non-synonymous homoplasies (36) in known resistance genes (35) and incorporated phenotype profiles for these isolates where available. This approach identified homoplastic mutations affecting codons 486 (*N* = 2) and 259 (*N* = 2) of the *gyrB* gene, although none of the four isolates with these mutations possessed phenotype data for fluoroquinolones (FLQs). Resistant isolates with substitutions in codon 486 can be found in the WHO catalog (35) but at a relatively low frequency. Homoplasies also occurred within codons 202 (*N* = 2) and 83 (*N* = 2) of the *thyA* gene, which is linked to PAS resistance (37). Phenotypic data were available for one isolate with each mutation, both of which were found to be resistant. We stress though that larger sample sizes will be needed to demonstrate robust associations between these markers and second-line drug resistance.

All RIF-resistant isolates which were predicted correctly were found to possess a resistance-associated variant within the RIF resistance determining region, indicating that the sensitivity of the GeneXpert assay would equal that of WGS data (0.70).

We found the frequency of putative resistance-conferring variants to be similar between TBM and PTB isolates (30.5% vs 24.9% for INH, 1.2% vs 4.7% for RIF, 3.0% vs 4.9% for EMB, 40.1% vs 36.4% for STR). We further verified that TBM isolates were dispersed throughout the phylogeny and were not clustered in monophyletic clades (Fig. S2), justifying their inclusion in the subsequent analysis of antibacterial resistance dynamics.

### Dynamics of drug resistance acquisition and transmission

**Fig 3:**
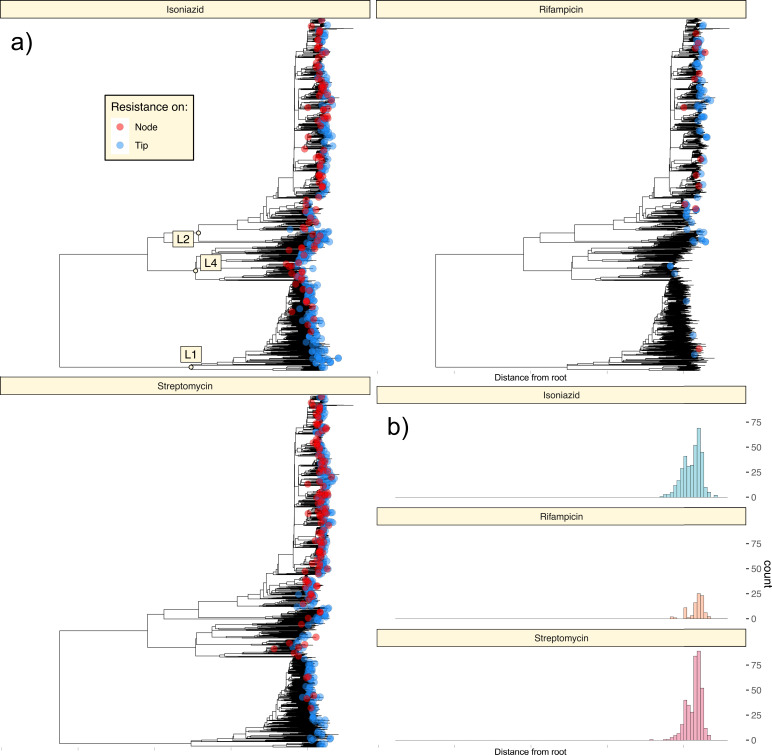
(a) Acquisition and transmission of mutations conferring resistance to three first-line drugs: INH, RIF, and STR. Biallelic SNPs present within the TB-Profiler catalog were mapped onto nodes of the phylogeny using SNPPar and labeled according to whether they arose on a tip/terminal branch (blue circles) or interior branch (red circles). (b) Histograms showing the distribution of resistance mutation emergence events relative to the root of the tree. X-axis scale of the histogram matches that of the above phylogeny.

We investigated the dynamics of drug resistance evolution within our collection to understand temporal and lineage-specific trends in drug resistance development. To do this, we used ancestral state reconstruction to map individual resistance mutation events to the phylogeny, to gauge the depth of mutations, and to differentiate between instances in which mutations map to nodes (Fig. 3a; red points) vs terminal branches (Fig. 3a; blue points).

Previous studies using similar methodologies class the former of these as cases of transmitted resistance and the latter as cases of acquired resistance (13, 17, 20, 38). We have remained cautious in our interpretation of these classifications, as our study samples only a small fraction of TB cases in HCMC, and, according to our participant recruitment inclusion criteria, the cases we analyze should not have received prior TB treatment (which is necessary for the classification of acquired resistance). We focus instead on the insight that these data give us regarding trends in the evolution of resistance between lineages and between drugs.

Across the phylogeny, we inferred 826 unique mutation events leading to INH, STR, or RIF resistance (EMB was not modeled due to the low positive predictive power of known variants, see Fig. 2a). We observed a trend in the order of resistance acquisition (Fig. 3), with INH and STR mutations arising earlier (closer to the root of the tree) than RIF mutations (Fig. 3a and b; median heights of 0.01285, 0.01291, and 0.01299 for INH, STR, and RIF variants, respectively; one-sided Mann-Whitney *U* tests, INH vs RIF: *P* = 1.38×10^−7^; STR vs RIF: *P* = 7.36×10^−7^).

To support this inference, we also considered the relative depth of mutations in branches leading to isolates with resistance to multiple drugs. This analysis revealed INH resistance mutations arise prior to RIF resistance mutations in the vast majority of instances when these mutations coincided (*N* = 53 INH first; *N* = 0 RIF first; *N* = 24 same branch; Fig. S4). In contrast to some prior studies (20, 38), STR resistance was often the first form of resistance to arise and preceded INH resistance on more occasions than it was succeeded by it (*N* = 17 INH first; *N* = 23 STR first; *N* = 158 same branch; Fig. S4).

We also assessed the ordering of resistance mutations for additional drugs for which resistance variants were associated with high positive predictive values, including fluoroquinolones (LEV and MXF), PAS, and DLM. We found fluoroquinolone resistance mutations to occur after INH mutations (*N* = 6 INH first; *N* = 0 FLQ first; *N* = 4 same branch; Fig. S4) and only on terminal branches (Fig. S3), illustrating the recent emergence of extensively drug resistant (XDR) TB within this population.

Alarmingly, we observed a single DLM resistance mutation which was relatively deeply rooted in the phylogeny, being transmitted to 26 isolates, and associated with phenotypic resistance in the 4 of the 5 of these isolates tested (Fig. S3). Contrary to some prior studies (38, 39), we did not identify early or widespread resistance to PAS via known genetic variants (*N* = 8 INH first; *N* = 1 PAS first; *N* = 4 same branch; Fig. S3 and S4).

Consistent with the relative ordering of resistance acquisition, drugs varied in the proportion of resistance mutations which can be mapped to internal nodes vs terminal branches of the phylogeny. A total of 63.6% (402/632) of isolates with INH resistance mutations were inferred to have inherited their resistance mutation from an unsampled ancestor, while the remainder were associated with mutations mapped to their terminal branch. A similar proportion was calculated when considering STR resistance variants (66.6%; 519/779), yet a lower figure for RIF (38.2%; 42/110). These results, while not allowing precise inferences of the rates of acquired vs transmitted resistance, illustrate the early emergence and ongoing circulation of INH- and STR-resistant TB in HCMC.

### Lineage-specific trends in drug resistance evolution

We observed clear lineage-specific trends in the dynamics of drug resistance evolution. Consistent with prior estimates of drug resistance rates across lineages (13, 16, 31, 32), we observed lineage 2 isolates to accumulate resistance-conferring mutations more frequently than isolates from other lineages. A total of 1.13% of all mutation events occurring along branches of the lineage 2 clade were associated with INH, STR, or RIF resistance: a higher figure than for lineages 1 and 4 (0.35% and 0.32%, respectively). When considering only isolates within sublineage L2.2.1, this figure rose to 1.27%.

We also found that a higher proportion of lineage 2 and sublineage 2.2.1 isolates with resistance mutations were inferred to have inherited those mutations (thus indicating direct evidence of transmission), relative to other lineages. For lineage 2, 74.6% (*N* = 347/465) of INH-resistant isolates inherited their resistance mutation, a higher figure than for lineages 1 (20%; *N* = 20/100) and 4 (52.2%; *N* = 35/67; χ^2^ test comparing proportions of isolates with resistance mutations mapping to nodes vs terminal branches in L2 vs non-L2 isolates; *P* < 2×10^−16^). Similar results were observed when considering STR resistance (L2, 71.9%, *N* = 487/677; L1, 15.9%, *N* = 11/69; L4, 63.6%, *N* = 21/33; *P* = 1.393 × 10^−15^). These figures were again more pronounced for sublineage 2.2.1, for which 77.9% (331/425) and 73.3% (470/641) of INH and STR mutations were inherited from a node.

We found lineages to display systematic tendencies to evolve resistance via mutations in certain genes. For this analysis, we compared isolates from lineages 1 and 2, which are the most numerous in our data set and which display the most extreme differences in phylogenetic characteristics and life history strategies (22, 40).

**Fig 4:**
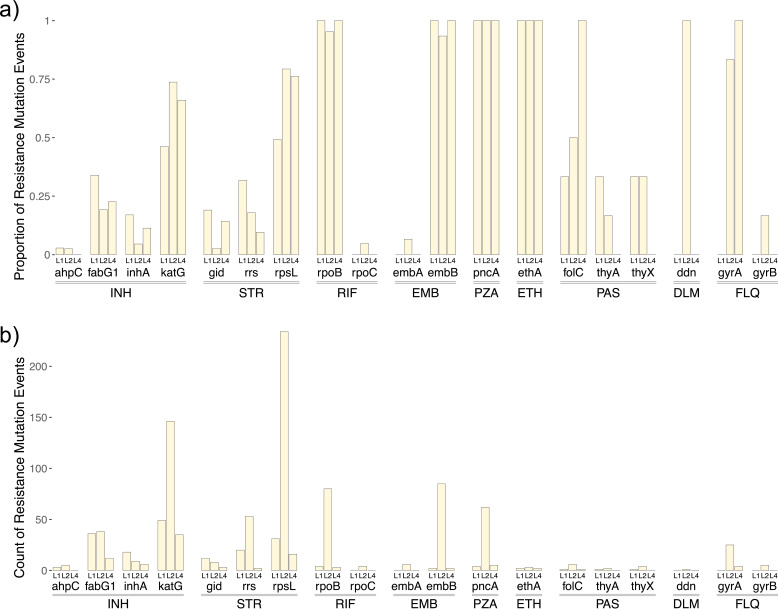
(a) Barplot showing the proportion of resistance mutation events (for a given drug) which arose within each gene (or gene promoter region) for lineages 1, 2, and 4. For example, the bar for *inhA* in L1 shows the proportion of all INH resistance mutations within the L1 tree which occur in the *inhA* gene. Genes are grouped by drug. (b) Barplot showing the number of times resistance-associated mutations within a given gene or promoter region arose among each of the lineage 1, 2, and 4 clades. Note that sample sizes for each lineage differ (L2, *N* = 1,615; L1, *N* = 649; L4, *N* = 275).

Among isolates from lineages 1 and 2 with STR resistance variants, mutations in the *rpsL* gene were more frequent among lineage 2 isolates (79.6% of STR-resistant variants in L2 found in the *rpsL* gene vs 49.2% for L1; χ^2^ test; *P* = 1.26 × 10^−6^; Fig. 4a), and *gid* mutations, among lineage 1 isolates (2.7% of STR-resistant isolates in L2 found in the *gid* gene vs 19.0% for L1; *P* = 1.495 × 10^−6^). Within the *rrs* gene, the C1472362T mutation arose more than twice as often in L1 than L2 (13 vs 6 mutations), whereas the A1472359C mutation arose more than 10 times as often in lineage 2 (3 vs 44 mutations).

Among lineage 1 and 2 isolates with INH resistance variants, *katG* mutations were more frequent in L2 isolates (accounting for 73.7% of INH mutations in L2 vs 46.2% in L1; *P* = 3.47 × 10^−6^), and *inhA* (4.5% in L2 vs 17.0% in L1; *P* = 6.25 × 10^−4^) and *fabG1* (19.2% in L2 vs 34.0% in L1; *P* = 0.00654) mutations were more frequent in L1 isolates (Fig. 4a). For all genes implicated in resistance to RIF, EMB, and FLQ, 95% or more of all resistance-associated variants occurred in the L2 clade (Fig. 4b).

When considering mutations within the TB-Profiler catalog recognized to play a compensatory role in drug resistance (41, 42), we found these mainly to be restricted to lineage 2 isolates. The *rpoC* mutations *rpoC*-Phe452Ser (*N* = 2), *rpoC*-Asp485Asn (*N* = 1), and *rpoC*-Leu527Val (*N* = 1) occurred only among lineage 2 isolates and only in the presence of *rpoB*-Ser450Leu mutations. No *rpoA* variants listed in the TB-Profiler catalog were documented in our data set. The *ahpC* promoter mutations *ahpC*_g-48a (*N* = 4), *ahpC*_c-81t (*N* = 1), *ahpC*_c-57t (*N* = 1), and *ahpC*_c-52a (*N* = 4) occurred both among lineage 1 and 2 isolates (*N* = 3 L1 and *N* = 7 L2). Six of the 10 isolates with *ahpC* mutations did not possess a *katG* mutation; however, only two of the five of these with phenotyping data were INH resistant, supporting the limited role of *ahpC* mutations in INH resistance alone [reviewed by reference (41)].

### Transmission dynamics

Finally, we investigated whether *Mtb* isolates with genotypic resistance were less likely to be transmitted than susceptible isolates. We are interested in this question, as resistance-conferring variants are typically associated with fitness costs in *Mtb* and other bacterial species (41, 43 – 45). Determining the potential of resistant strains to transmit to new human hosts will therefore be relevant in determining future drug resistance trajectories worldwide.

To gauge transmission success, we used the time-scaled haplotypic density (THD) statistic: a quantitative measure of the tendency of a strain to transmit, based on the density of coalescence events within its recent evolutionary history (46). Similar to prior studies (39, 46, 47), we used this metric as a response variable in linear regression, incorporating sublineage [either L1, L2.2.1, and L2 isolates excluding L2.2.1 (L2(x2.2.1)) or L4] and drug resistance status as covariates and modeling an interaction between sublineage and resistance status. The resistance statuses considered were pan-susceptible (susceptible to INH, RIF, and STR), INH mono-resistant (resistant to INH and susceptible to RIF and STR), STR mono-resistant (resistant to STR and susceptible to RIF and INH), INH and STR resistant (resistant to INH and STR, but lacking RIF resistance), and MDR with STR resistance (resistant to INH, RIF, and STR). The small number of isolates which did not fit within these categories (i.e., MDR isolates lacking STR resistance; *N* = 5) were not considered, nor were isolates with inconsistent phenotype profiles.

**Fig 5:**
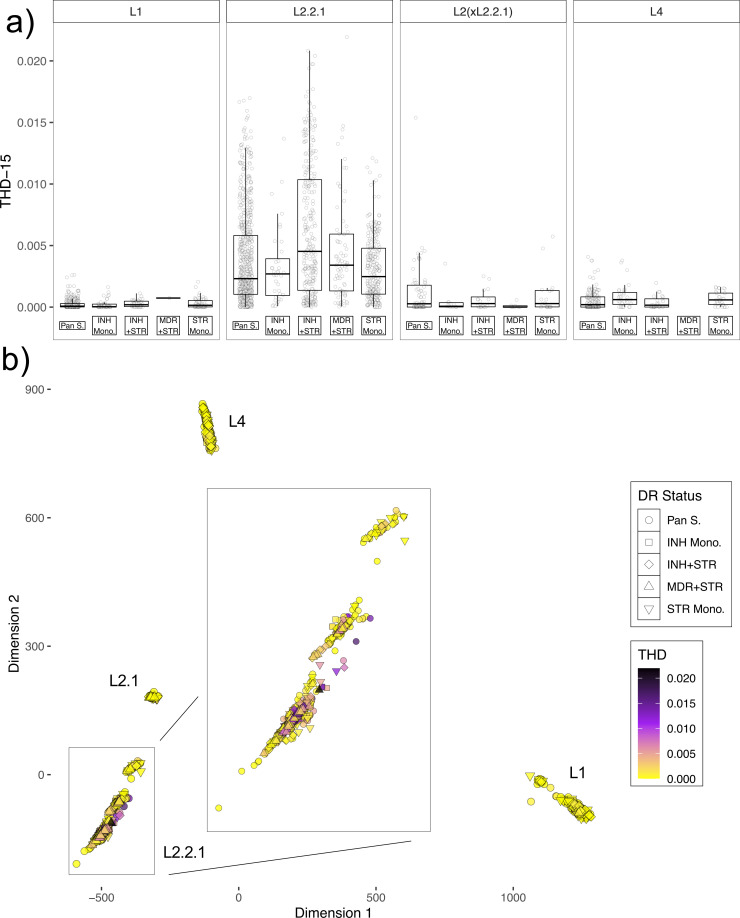
(a) Scatterplots with overlaid boxplots showing the distribution of THD values (when applying a 15-year threshold) for isolates with five different drug resistance profiles (either Pan_S, INH_Mono, STR_Mono, INH + STR, MDR + STR). Isolates were also stratified on the basis of lineage or sublineage. (b) MDS plot of all isolates included in the THD analysis, with point shape indicating drug resistance profile, and fill color indicating THD value. An inset magnifying the L2.2.1 cluster was added to aid visibility.

Fitting this linear model revealed an influence of lineage designation on THD statistic, with sublineage 2.2.1 possessing a clear enrichment of THD relative to L1 (*P* < 2 × 10^−16^; Fig. 5a and b). A non-significant result was obtained for lineage 4 (*P* = 0.28), and a relatively weak but significant result, for L2(xL2.2.1) (*P* = 0.01). When considering drug resistance status, the combination of INH and STR resistance was associated with increased THD values relative to pan-susceptible isolates but only for isolates within sublineage 2.2.1 (*P* = 2.8 × 10^−5^; Fig. 5a). All other combinations of lineage and drug resistance status yielded non-significant *P*-values (Fig. 5a). The effect of INH + STR resistance on THD in L2.2.1 isolates was also highly significant when incorporating multidimensional scaling (MDS) components as covariates in linear regression (*P* = 2.14 × 10^−8^), suggesting that substructure within the L2.2.1 sublineage was not driving the association. Replicating these analyses with different THD thresholds supported these inferences (Fig. S5 and S6).

## DISCUSSION

### High prevalence of phenotypic drug resistance in *Mtb* isolates from HCMC

Our investigation has supported prior reports (48, 49) of the high rate of resistance to several first-line drugs, in addition to MDR, within a Vietnamese *Mtb* collection. The frequencies of resistant isolates found here are similar to a recent national survey of Vietnam (49), which also reports high rates of STR (27.4%) and INH (18.9%) resistance and lower rates of RIF (4.1%) and EMB (3.4%) resistance among new TB cases. When considered globally, these individual first-line drug resistance values were higher than what has been reported in parts of Africa (50 – 52), East Asia (53, 54), and South Asia (55) but lower than a South American cohort (56). The second-line drug resistance rates that we observe are higher than what was reported in the small number of studies which survey second-line resistance in new TB cases (54, 56, 57).

### Concordance between drug resistance phenotype and genotype-based predictions

Our analysis has also provided an assessment of the accuracy of genotype-based drug resistance prediction within a Vietnamese population. Contextualizing the results that we obtained is difficult, as prior studies have typically relied on much smaller sample sizes (58 – 60), have not reported data for the same subset of drugs analyzed here (8), or have systematically removed isolates with uncharacterized mutations in target genes (5). We contrast our results here with those of Mahe et al. (34) who analyze a large sample (>6,000 isolates) using the same prediction catalog and methodology.

Our sensitivity values for INH and STR prediction are comparable with those of Mahe et al. (34) (0.89 and 0.77, respectively), and specificity values for all four first-line drugs are equal to or higher than those of Mahe et al. (34) (INH = 0.97; RIF = 0.98; EMB = 0.93; STR = 0.91). Our sensitivity values for the two drugs with the lowest levels of phenotypic resistance, RIF and EMB, are, however, lower than those of Mahe et al. (34) (0.91 and 0.93, respectively). Also, our ability to predict resistance to second-line drugs was considerably lower than Mahe et al. (34), who report sensitivity values of 0.92, 0.92 and 0.89 for KAN, AMI, and aggregated fluoroquinolones, respectively.

We conclude that prediction accuracies for the two drugs with the highest rates of resistance, INH and STR, are comparable with global benchmarking standards. We also note that the frequency distributions of markers explaining resistance in correctly predicted isolates are consistent with a recent survey of the Vietnamese population (21) and the recently published WHO catalog (35). In all instances, the most commonly reported resistance-conferring variants in our data set were also among the most frequent in the catalog collated by the WHO (35).

A future avenue of research may involve investigating the genetic basis of resistance in the high number of EMB-resistant isolates which were predicted to be susceptible using the TB-Profiler catalog (*N* = 42/75, 56%). It is clear that large studies correlating genotypic predictions of resistance with clinical outcomes are essential to improve the accuracy of genotypic resistance prediction and to resolve discrepancies between phenotypic and genotypic prediction, especially for the antimycobacterial drugs with poorly characterized resistance mechanisms.

### Acquisition and transmission of drug resistance amongst L2.2.1 isolates

This investigation has explored the dynamics of *Mtb* drug resistance evolution in a high-burden Southeast Asian setting. Analysis of cohorts from other geographical regions has alternately emphasized the roles of transmission (13, 16, 19) or *de novo* acquisition (17, 18, 20) in explaining rates of drug resistance. For instance, Wollenberg et al. (13) class 90% of INH-resistant isolates as transmitted in their cohort from Belarus, while Ektefaie et al. (20) class around 40% as transmitted in their global cohort. We report an intermediate figure for INH-resistant cases (63.6% of isolates with resistance mutations inherited them from nodes) but caution that these figures are highly dependent on the sampling regime.

Importantly, we find that drug resistance burden in Vietnam is predominantly driven by Beijing lineage 2.2.1 isolates, which accumulate resistance-conferring mutations more frequently than other isolates and display a tendency to transmit resistance between hosts. While our THD analysis suggested that the possession of drug resistance does not act to impede the transmission of isolates from any lineage, it did support the higher rates of transmission for sublineage 2.2.1 isolates across all drug resistance categories. Furthermore, among lineage 2.2.1 isolates, the combined presence of INH and STR resistance was found to render isolates more transmissible. These data, therefore, highlight an additional danger associated with the L2.2.1 sublineage, which is gradually supplanting endemic lineage 1 isolates within Vietnam (22).

### Evolutionary pathways to drug resistance differ between *Mtb* lineages

We were also interested to find that *Mtb* lineages displayed tendencies to evolve resistance via mutations in certain genes and promoter regions. Specifically, we found STR resistance to be more frequently mediated by *rpsL* mutations in lineage 2 isolates relative to lineage 1 and INH to be more typically mediated via *inhA* and *fabG1* mutations in lineage 1. A prior study associated *katG*-Ser315Thr mutations with comparatively high-level INH resistance in lineage 2 isolates compared to lineage 1 and *inhA* promoter mutations with high levels of resistance in lineage 1 (61), a topic reviewed in reference 62. Our analysis may provide an additional line of support for these inferences, by demonstrating that mutations in these genes have evolved more frequently in each respective lineage within a naturally evolving population of *Mtb*.

We also observed a similar pattern involving the *rpsL* and *gid* genes implicated in STR resistance. Previous studies have documented high frequencies of *rpsL* mutations in isolates from lineage 2 (63, 64) and differences in the resistance level conferred by mutations in the *rpsL* and *gid* genes (64). This analysis highlights the need for future research into the interaction between lineage and resistance levels in the context of STR resistance.

### Limitations of this analysis

A caveat which must be applied to our findings is that our phylogenetic methods are unable to model the evolution of resistance in isolates for which their phenotype cannot be explained by markers in the TB-Profiler catalog. It is possible that inferences surrounding the evolution of RIF resistance, for instance, may change with the identification of more resistance-conferring markers. Resistance explained by indels is similarly unable to be modeled using our methods; however, this class of variant represents a small fraction of our data set.

### Implications for TB control in Vietnam

In addition to elucidating dynamics of drug resistance evolution within our collection, we have also established that resistance to INH and STR arose earlier, on average, than resistance to RIF and is now more widespread within the Vietnamese population. The emergence of STR resistance as the first form of drug resistance is inconsistent with studies of other global regions (38, 39) and may be attributed its introduction in the early 1950s in Vietnam (65). The high levels of STR resistance and propensity for lineage 2 isolates to develop it support the decision to remove this drug from first-line treatment regimes.

The inference of early INH resistance evolution is consistent with prior studies and mimics results obtained from a South African population (18) and two surveys of global *Mtb* isolates (17, 20). We conclude, as do Manson et al. (17), that a rapid assay for INH resistance will allow the detection of “pre-MDR” TB and offer the high number of patients with INH-resistant TB treatment options which include other drugs. Unlike the cohort analyzed by Manson et al. (17), however, we find that a high percentage of RIF resistance isolates are susceptible to INH (20.7%). This finding further supports the utility of an INH resistance reflex test, which may be applied after a patient tests positive for RIF resistance using the MTB/RIF Xpert test. Several WHO-approved tests for INH resistance could be implemented (66). Application of such INH resistance tests will allow the 20% of RIF-resistant cases which remain susceptible to INH to be treated with an INH-containing regimen.

Finally, the high rates of resistance to a number of second-line drugs highlight the utility of WGS-based individualized therapy for drug-resistant TB in Vietnam. The poor sensitivity values calculated, however, stress the need to develop a greater understanding of the genetic variants implicated in resistance to these drugs.

## MATERIALS AND METHODS

### Study population

Bacterial isolates (*N* = 2,619) from patients with PTB (*N* = 2,446) and TBM (*N* = 173) were collected as part of larger clinical studies (23 – 25). Patients with PTB were defined as HIV-negative adults (>18 years) with sputum culture positive for *M. tuberculosis*. Isolates (*N* = 1,654) were collected in eight district TB units (DTUs) in HCMC, Vietnam between December 2008 and July 2011 (25). A further 792 isolates from PTB patients were similarly collected at these DTUs in HCMC as an extension to this clinical study, from 2011 to 2013. Patients with TBM were defined as HIV-negative patients, >15 years old, with cerebral spinal fluid culture positive for *Mtb* and were recruited into two clinical trials conducted at the Hospital for Tropical Diseases and Pham Ngoc Thach Hospital for TB and Lung Diseases in HCMC (23, 24). Sixty-two were collected between 2001 and 2003 (24) and 111 were collected between 2011 and 2015 (23), with a total of 173 isolates from TBM patients included in this genomic study.

### Phenotypic drug susceptibility testing (DST)

Phenotypic DST was performed using two techniques. In the first method, isolates were subcultured in MGIT for phenotypic DST on the first-line drugs, INH 0.1 µg/mL; STR 1.0 µg/mL; RIF 1.0 µg/mL; EMB 5.0 µg/mL, using the BACTEC MGIT 960 SIRE Kit (Becton Dickinson) according to the manufacturer’s instructions. The second method used the UKMYC5 plate designed by the CRyPTIC consortium which enables minimum inhibitory concentration (MIC) measurement for 14 different anti-tuberculosis compounds. The UKMTC5 MIC plate method was used as described in Rancoita et al. (67). The critical concentrations used to determine drug resistance were bedaquiline 1.0 µg/mL, KAN 2.5 µg/mL, ETH 5.0 µg/mL, AMI 1.0 µg/mL, EMB 5.0 µg/mL, INH 0.1 µg/mL, LEV 1.0 µg/mL, MXF 0.5 µg/mL, DLM 0.12 µg/mL, LZD 1.0 µg/mL, CFZ 1.0 µg/mL, RIF 1.0 µg/mL, RFB 1.0 µg/mL, and PAS 4.0 µg/mL.

### DNA extraction and sequencing

Lowenstein-Jensen media were used to subculture isolates at the Oxford University Clinical Research Unit, Vietnam, prior to DNA extraction using the cetyl trimethylammonium bromide extraction protocol as described previously (68). DNA was shipped to the University of Melbourne (*N* = 1,827) and the National University of Singapore (*N* = 792) for whole genome sequencing. At the Genome Institute of Singapore (GIS), genomic DNA was first quantified by Picogreen assay, followed by shearing using the Covaris. Library preparation was done using a commercially available kit, NEBNext Ultra DNA Library Prep Kit for Illumina following the manufacturer’s protocol. The quality of the libraries were QC via LabChip GX or Agilent D1000 ScreenTape before pooling. After pooling, the pooled library was QC’d using Agilent high-sensitivity DNA kit and KAPA quantification before sequencing on the Illumina HiSeq 4000 (Illumina, San Diego).

### *Mtb* genome data and SNP calling

*Mtb* genome data from a subset of PTB patients have been previously described and is denoted here as the “published subset” (22). The complete genome collection is denoted as the HCMC PTB/TBM genome data set. Variant calling for *Mtb* isolates was carried out using the RedDog pipeline V1beta.11 (https://github.com/katholt/RedDog) with default settings, which uses BowTie (69) for read mapping and Samtools (70) for variant calling. After variant calling, samples for which less than 90% of their reads mapped to the *Mtb* H37Rv reference genome (NC_000962.3) and with high proportions of heterozygous sites were removed. Variants called in repetitive regions, as defined by Holt et al. (22), were also removed. *Mtb* lineages and sublineages were assigned using fast-lineage-caller (27) on per-sample vcf files, using the scheme of Coll et al. (71). Sequence reads from an isolate belonging to lineage 5 were also incorporated into the above variant calling pipeline as an outgroup for all subsequent phylogenetic analysis.

### Drug resistance prediction using whole genome sequencing data

Whole genome sequencing-based resistant prediction was performed with TB-Profiler v4.2.0 (33) using the most up-to-date database available (16 February 2022). Read data were screened for mutations associated with resistance to four first-line drugs (INH, RIF, EMB, and STR) and second-line drugs for which phenotypes were available and which are covered by the TB-Profiler catalog. For context, the TB-Profiler algorithm outputs a prediction of “resistant” if an isolate possesses any of the variants listed in the catalog for that particular drug. Sensitivity values were calculated as described by Mahe et al. (34) both when considering all isolates and when restricting to isolates from a particular lineage only.

### Phylogenomic analysis

We inferred a phylogeny from 2,542 *Mtb* genomes using RAxML v8.2 (72), using a GTR model of nucleotide substitution. Ancestral state reconstruction was then performed using SNPPar v1.0 (73) with default settings. Biallelic SNPs conferring drug resistance were defined according to the TB-Profiler and extracted from the SNPPar output. We did not consider variants which SNPPar was unable to map to the phylogeny, including indels, and sites which were called by TB-Profiler, but not by the Reddog pipeline. All downstream analyses of tree diversity metrics were carried out using custom Unix and R scripts (74) and the R packages ape v5.6.2 (75), treeio v1.18.1 (76), and phangorn v2.8.1 (77).

Similar to prior studies (13, 17, 20, 38), we explored the relative depth and ordering of resistance mutations for insight into antimicrobial resistance (AMR) transmission dynamics. We distinguished between resistance mutations which could be mapped to nodes vs terminal branches of the phylogeny and plotted these as red and blue points, respectively (Fig. 3a), on the end of the branch on which they arose.

After applying these classifications, we calculated metrics, including the proportion of isolates with resistance mutations for a given drug which were inherited from a node vs a tip. For the small number of isolates (*N* = 5) across the phylogeny which developed resistance-conferring variants on their terminal branch but which had already descended from a resistant node, we counted among the isolates inheriting resistance from a node. For the analysis which describes the ordering of resistance mutations for pairs of drugs, we applied custom Unix scripts to the output of SNPPar.

We excluded EMB from the above analyses as our ability to accurately model transmission dynamics would be limited due to the low predictive power of EMB resistance variants (59.8% of isolates with putative EMB resistance variants were phenotypically susceptible; Fig. 2a), in addition to the low sensitivity demonstrated previously (Fig. 2a). We also excluded ETH for the same reasons and pyrazinamide (PZA) due to the lack of phenotyping data for this drug.

For our description of the occurrence of compensatory mutations, we referred to the studies of Alame-Emane et al. (41) and Napier et al. (42) and considered TB-Profiler mutations in the *rpoA*, *rpoC*, and *ahpC* genes as compensatory. For our analysis of homoplastic mutations in second-line drug resistance associated genes, we identified recurrent mutations from the SNPPar output and restricted these to the resistance genes listed in the WHO catalog (35).

### Transmission inference

To compare the transmissibility of isolates with various drug resistance profiles, we calculated the THD statistic (46, 47). THD was computed using the “thd” package in R (46) on a pairwise matrix of SNP distances between isolates. The parameters used for the THD calculation included a mutation rate of 1.1 × 10^−7^ (7), a timescale parameter of 15 years, and 4,013,003 as the number of non-masked positions in the *Mtb* genome. Isolates were classified according to their sublineage [either L1, L2.2.1, L2(x2.2.1), or L4] and drug resistance status [either pan-susceptible (Pan_S), INH mono-resistant (INH_Mono), STR mono-resistant (STR_Mono), resistant to INH and STR (INH + STR), or MDR with STR resistance (MDR + STR)] as described in the Main Text. In order to incorporate all isolates, genotyping data were used to determine drug resistance profile, although we identified and removed any isolates with discordant phenotype profiles.

We performed linear regression using the lm() function of the stats package of R (R Core Team, 2021), modeling the THD statistic as the continuous response variable, and incorporating sublineage and drug resistance status as covariates. Pan_S and L1 were used as the reference levels.

To account for population structure, we performed MDS using the “cmdscale” function of the stats package of R (74). We ran MDS on the entire data set (all isolates) and after subsetting the data set to isolates from sublineage 2.2.1. To test the association between THD and drug resistance status in L2.2.1 isolates, we ran an additional regression which incorporated the top 12 MDS components of the L2.2.1 MDS as covariates. We also replicated this protocol using THD timescales of 10 and 20 years (Fig. S5 and S6).

### Statistical analysis

Analyses were done using R version 4.1.1, and two-sided *P* < 0.05 was considered statistically significant. The overall lineage distribution of *Mtb* isolates across gender, clinical phenotype, and resistance to the first and second anti-TB drugs was presented as number and percentage (%). Pearson’s χ^2^ test was performed to investigate the relationship between categorical variables, including lineages and other clinical characteristics, naming gender, clinical phenotype, and drug resistance. It was also utilized to assess the relationship between rates of drug resistance and lineages, as well as resistance and transmission ability. Mann-Whitney *U* test was employed to test the difference for non-normally distributed variables among lineages, including count of total mutations relative to the number of resistance-conferring mutations and lengths of terminal branches leading to resistance. The association between age and lineage (lineages 1 and 2) was assessed using linear regression. To investigate the prediction accuracy for drug resistance using WGS data, we calculated sensitivity, defined as the proportion of resistant isolates correctly predicted; specificity, defined as the proportion of susceptible isolates correctly predicted; and PPV and negative predictive value.

## Data Availability

Previously published *Mtb* genomic sequences are available from the European Nucleotide Archive, under accession number PRJNA355614. Newly generated sequences are also available, under accession number PRJNA1028637.

## SUPPLEMENTAL MATERIAL

Supplemental material spectrum.02562-23-s0001.docx Fig. S1 to S6 and Table S1.

ASM does not own the copyrights to Supplemental Material that may be linked to, or accessed through, an article. The authors have granted ASM a non-exclusive, world-wide license to publish the Supplemental Material files. Please contact the corresponding author directly for reuse.
