# Supplementary material for: Evolution and transmission of antibiotic resistance is driven by Beijing lineage Mycobacterium tuberculosis in Vietnam
Source: Microbiol Spectr. 2023 Nov 16;11(6):e02562-23. doi: 10.1128/spectrum.02562-23 (PMC10714959; doi:10.1128/spectrum.02562-23)
Supplement: Supplemental material — Fig. S1 to S6 and Table S1. [file spectrum.02562-23-s0001.docx]

**
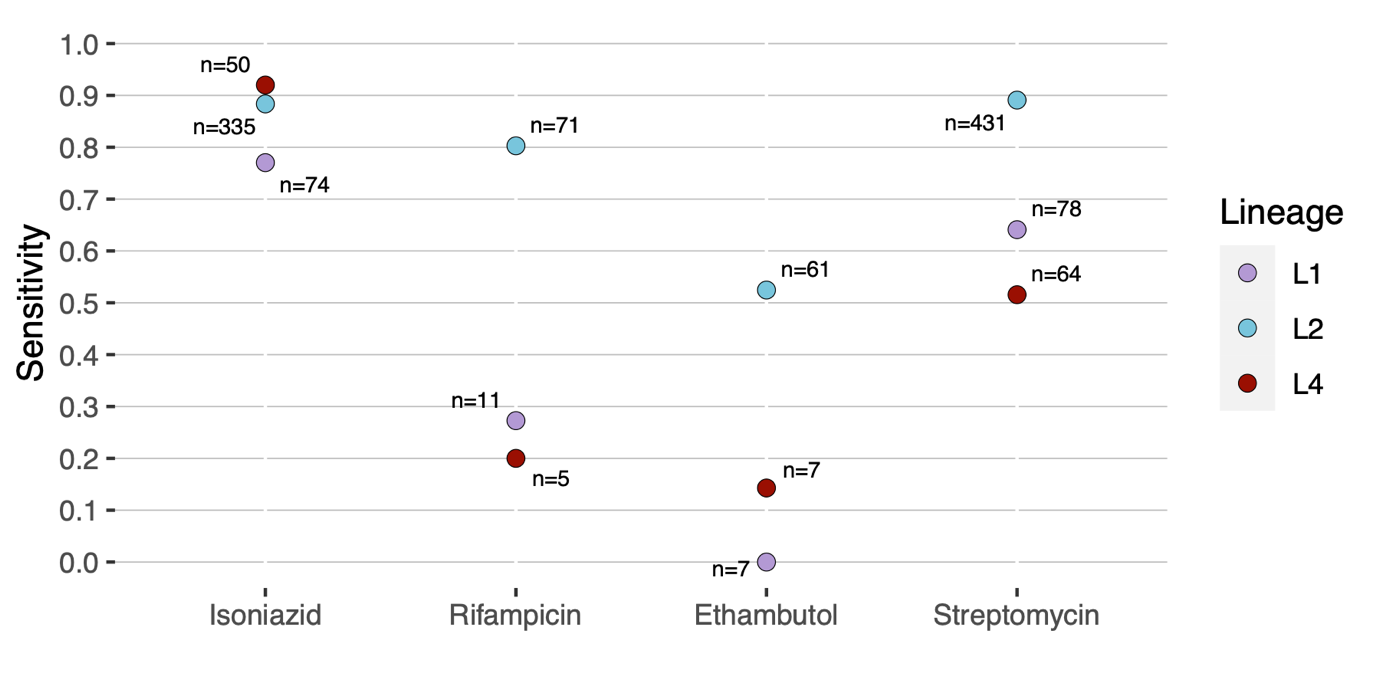
**

**Figure S1. Sensitivity values for drug resistance prediction from *Mtb* sequence data for the four first line drugs with phenotypic DST data. Sensitivity was measured individually for each of the three main *Mtb* lineages (L1, 2 and 4). The number of resistant isolates for each lineage/drug combination is plotted next to each point.**

**Figure S2. Distribution of TBM across the phylogeny of N=2,542 *Mtb* isolates from HCMC, Vietnam.**

| **Drug** | **Sensitivity** | **Number of resistant isolates** |
| --- | --- | --- |
| BDQ | 0 | 1 |
| DLM | 0.44 | 9 |
| CFZ | 0 | 7 |

**Table S1. Sensitivity values for second line drugs with fewer than ten resistant isolates**

**
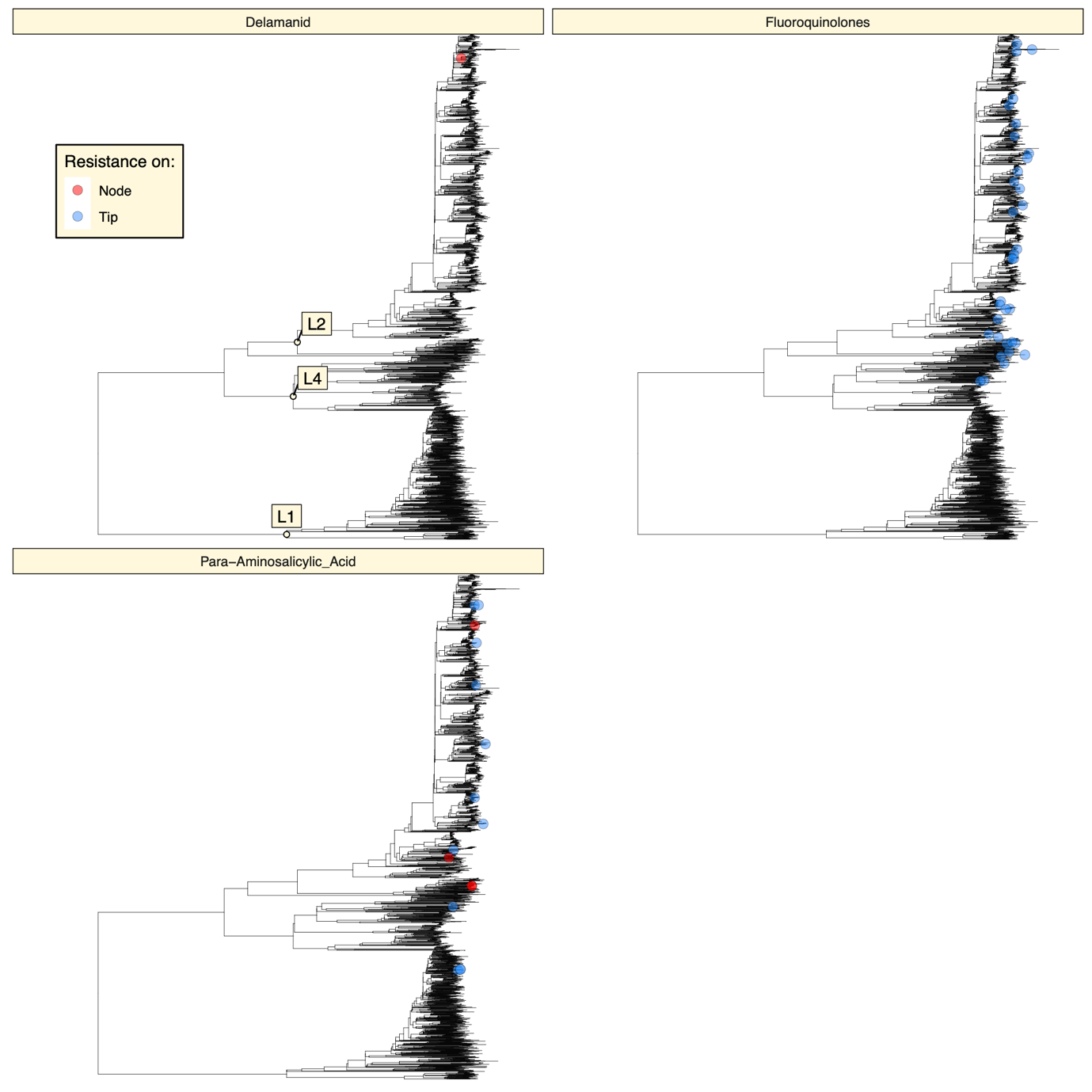
**

**Figure S3. Phylogeny of *Mtb* isolates from HCMC with mutations associated with second line drug resistance (defined according to TB-Profiler) marked on the branches on which they were inferred to occur. Point colour denotes whether these mutations map to a node (red) versus a terminal branch (blue).**

**Figure S4. Heatmap showing the relative ordering of mutations associated with resistance to various combinations of drugs. The numbers in brackets indicate the total number of times resistance mutations for that pair of drugs co-occurred. For instance, INH and STR resistance occurred together 198 times. On 17 occasions, INH arose first, and on 23 occasions STR arose first. On all remaining occasions, these mutations arose on the same branch. Colour gradient shows the proportion of all co-occurrence events where resistance to that drug arose first. (FLQ = Fluoroquinolone).**

**
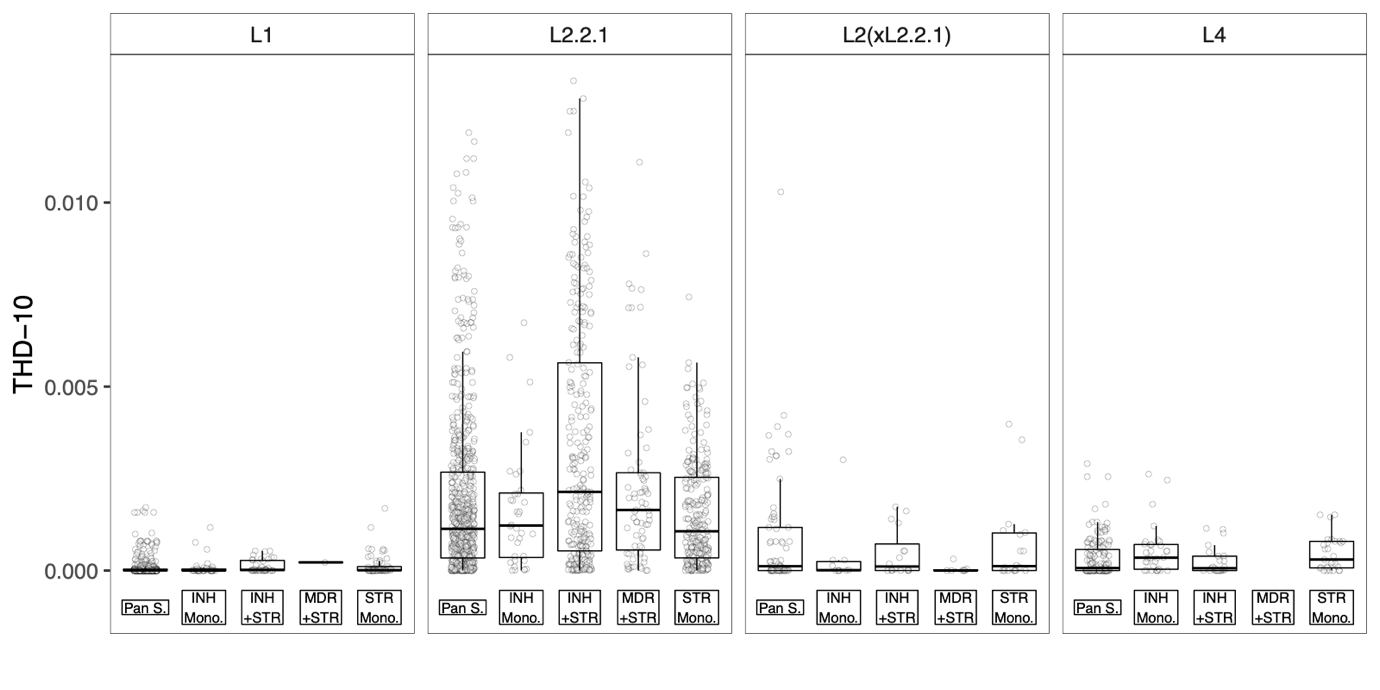
**

**Figure S5. Scatterplots showing the distribution of THD values (when applying a 10 year threshold) for isolates with five different drug resistance profiles (either Pan_S, INH_Mono, STR_Mono, INH+STR, MDR+STR). Isolates were also stratified on the basis of lineage or sublineage.**

**
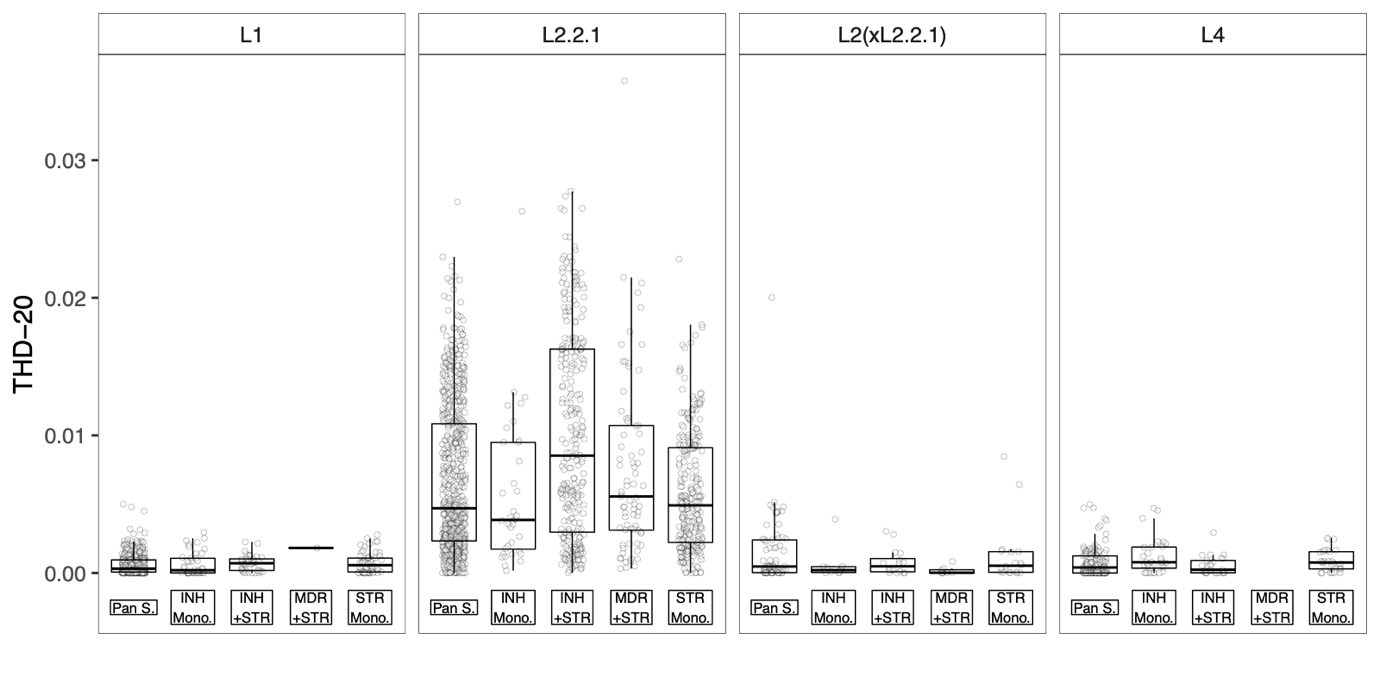
**

**Figure S6. Scatterplots showing the distribution of THD values (when applying a 20 year threshold) for isolates with five different drug resistance profiles (either Pan_S, INH_Mono, STR_Mono, INH+STR, MDR+STR). Isolates were also stratified on the basis of lineage or sublineage.**
